# Interpersonal neural synchrony when predicting others’ actions during a game of rock-paper-scissors

**DOI:** 10.1038/s41598-022-16956-z

**Published:** 2022-07-28

**Authors:** E. Kayhan, T. Nguyen, D. Matthes, M. Langeloh, C. Michel, J. Jiang, S. Hoehl

**Affiliations:** 1 Department of Developmental Psychology, University of Potsdam, Potsdam, Germany; 2 Max Planck Institute for Human Cognitive and Brain Sciences, Leipzig, Germany; 3 Faculty of Psychology, University of Vienna, Vienna, Austria; 4 Italian Institute of Technology (IIT) – Center for Life Nano Science, Rome, Italy; 5 Laboratory for Biosignal Processing, Leipzig University of Applied Sciences, Leipzig, Germany; 6 Institute of Psychology, Heidelberg University, Heidelberg, Germany; 7 Faculty of Education, Leipzig University, Leipzig, Germany; 8 Departments of Pediatrics and Psychiatry, Carver College of Medicine, University of Iowa, Iowa City, IA 52242 USA

**Keywords:** Cooperation, Empathy

## Abstract

As members of a social species, we spend most of our time interacting with others. In interactions, we tend to mutually align our behavior and brain responses to communicate more effectively. In a semi-computerized version of the Rock-Paper-Scissors game, we investigated whether people show enhanced interpersonal neural synchronization when making explicit predictions about others’ actions. Across four experimental conditions, we measured the dynamic brain activity using the functional near-infrared spectroscopy (fNIRS) hyperscanning method. Results showed that interpersonal neural synchrony was enhanced when participants played the game together as they would do in real life in comparison to when they played the game on their own. We found no evidence of increased neural synchrony when participants made explicit predictions about others’ actions. Hence, neural synchrony may depend on mutual natural interaction rather than an explicit prediction strategy. This study is important, as it examines one of the presumed functions of neural synchronization namely facilitating predictions.

## Introduction

We, as humans, spend most of our time interacting with others. When interacting, we tend to align our behavior, emotional expressions, and neural responses for effective communication. Despite being a fundamental aspect of our lives, the neural underpinnings of interpersonal synchrony have only recently gained interest among social neuroscientists. In this study, we investigated whether interaction partners show increased neural synchrony when they were asked to make explicit predictions about others’ actions as compared to implicit predictions while interacting freely as well as when they did not interact with one another. Using a Rock-Paper-Scissors game, we aimed to create a naturalistic yet structured context in which we measured the dynamic brain activity during the interaction using dual-functional near-infrared spectroscopy (fNIRS).

Dynamic mutual alignment underlies social interactions. The reciprocal exchange of information and experiences renders interactions social, which occurs even in the absence of shared goals^1^. In a competitive situation, for example, interacting agents might align their internal models and actions to solve a conflict or gain an advantage. Moreover, individuals tend to align their actions even if this outcome is not desirable for optimal performance^2^. Relatedly, it has been argued that interpersonal synchronization (i.e., coherence) might play a functional role in nonverbal communication such as coordination of motor behavior to make social interactions more efficient^3,4^.

There is a growing line of empirical evidence showing that individuals reciprocally adjust their behavior and neurophysiological processes during social interactions^5–9^. In their pioneering work, Cui et al. (2012) used fNIRS hyperscanning to measure neural coherence between two individuals as they played a computer-based game in which players either competed or cooperated with each other to win points. Data revealed increased neural coherence across frontal regions during cooperation as compared to competition. Moreover, neural coherence in the cooperation game positively correlated with the performance during the game^10^. Whereas some studies provided further evidence for enhanced neural coherence during cooperative interactions^8,10–13^, others showed that neural synchronization between interaction partners increased during competition^6,8^. For example, Cho et al. (2020) asked participants to control a cursor together and move it towards one of three designated targets either with the goal of competing or cooperating with each other. Results indicated higher early low beta band synchronization between dyads in the competition condition as compared to the cooperation condition^5^. Overall, these findings show that increased interpersonal neural synchrony emerges during social exchanges even in the absence of a mutual goal^1^.

Theoretical work suggested that during effective interactions the brain activity of two or more individuals would be aligned resulting in *generalized synchronization*^14,15^*.* In episodes of high neural synchronization, one would assume that the internal models of the interacting individuals are aligned such that they lead to similar predictions and actions^16^. For example, in a dialogue, when the listener predicts what the speaker will say next, generalized synchrony or neural alignment would emerge where the interlocutors’ brains entrain to one another, mediated through the speech signals^17,18^.

It should be noted that generalized synchronization is considered to be a dynamic phenomenon evolving over the course of the interaction as communicators construct a shared dynamic narrative. There has been emerging evidence supporting the idea that neural synchronization increases over the course of one-to-one interactions, when mutual engagement and turn-taking is high^19^. Building upon this idea, others argue that individuals align their internal models and behavior, as it is computationally more efficient^3^. In other words, the alignment of internal models, hence actions, functions as an optimization principle reducing the cost of processing social information^4^.

Empirical studies in the field of hyperscanning largely focused on cooperative and competitive situations in naturalistic interactions. Moreover, although theoretical accounts have been proposed to explain how neural synchrony might optimize processing of information in social contexts via predictions, empirical evidence on whether and how predicting others’ actions modulates neural synchronization in humans is lacking. To move the hyperscanning field forward, we need empirical study designs which directly test the role of predictions in the context of competitive and cooperative natural interactions^20^.

In a semi-computerized version of the Rock-Paper-Scissors game, we investigated the dynamic brain activity of participants when they made explicit and implicit predictions about the other player’s actions as well as when they played the game on their own (i.e., no predictions). In the *Free Play* condition, participants played the game as they would do in real life where they implicitly predicted the other player’s actions or not. In other words, it might be that although the participants were not explicitly instructed to predict, that is, make an educated guess about the other player’s action in each round, some participants might nevertheless have implicitly used a prediction strategy instead of playing randomly. In the *Prediction-Different Action* condition, participants were asked to explicitly predict the other player’s move. That is, participants were required to apply an explicit prediction strategy. To create a competitive situation, participants won points when they performed the action that would win against the predicted action (e.g. prediction rock, action paper). In the *Prediction-Same Action* condition, the game rules changed. Here, both participants won when they predicted and performed the same action as the other player (e.g. prediction rock, action rock). Therefore, a collaborative performance was required. In the *Control* condition, participants performed one of the three actions on their own, thus, no prediction strategy was required.

The objective of this study was to test whether neural synchronization is enhanced when making explicit predictions about others’ actions. We reasoned that this would allow us to provide empirical evidence on one of the presumed functions of neural synchronization namely facilitating predictions^21^. We hypothesized that there will be enhanced neural synchronization when participants try to actively and explicitly predict the other player’s actions. Because participants were asked to perform the same action to win in the *Prediction-Same Action* condition, we expected to see highest neural synchrony in this condition (i.e., collaboration). We expected to observe the second highest neural synchronization in the *Prediction-Different Action* condition representing a competitive situation. In the *Free Play* condition, we expected to observe lower levels of neural synchrony (i.e., the third highest coherence) as compared to the two explicit prediction conditions, as participants may have implicitly predicted the action of their partner when playing the game or not. Finally, we hypothesized to observe the lowest levels of neural synchrony in the *Control* condition as compared to all three interaction conditions as interpersonal synchrony is expected to arise during mutual exchanges. The pre-registration for the study is available at https://aspredicted.org/blind.php?x=5s4w6j.

We focused on frontal and temporo-parietal regions and calculated the wavelet transform coherence (WTC) as a measure of neural coherence^11,19,22^. Because the dorsolateral prefrontal cortex (DLPFC) has been functionally linked to the top-down control of cognitive processes, which has been observed in natural interactions such as verbal conversations, we chose to examine neural coherence in both left and right DLPFC^11,19^. Moreover, we examined activation in the temporo-parietal junction (TPJ) given its associations with mentalization processes, which is crucial in predicting others’ intentions and actions^23,24^, as well as with interpersonal synchrony^11,19,22,25^.

## Method

### Participants

Reporting effect sizes is only recently gaining momentum in fNIRS hyperscanning studies. For example, some studies report large effect sizes in verbally mediated cooperation tasks (e.g.^26,27^). Because the tasks used in our study did not include verbal communication, we opted for a medium effect size. According to a power-analysis, to detect an effect with an effect size of 0.3 with a power of 0.95, we needed to test a minimum of 22 dyads (G*Power, version 3.1.9.7). We recruited 32 (20 female, M = 23.8 years, SD = 2.8 years, age range: 18–30 years) right-handed, same-sex dyads (i.e., 64 individuals) from a database of volunteers. One dyad was excluded from the sample due to a technical error. The participants did not know each other prior to the experiment. The participants reported no prior psychiatric or neurological treatment. The research was performed in accordance with the Declaration of Helsinki. All participants gave written informed consent for the study. The procedures were approved by the Ethics Committee of the Max Planck Institute for Human Cognitive and Brain Sciences. The participants received a monetary reward for their participation.

### Experimental task and design

We balanced experimental control and ecological validity within one design using a semi-computerized version of the Rock-Paper-Scissors game. In each game, participants could win, lose, or tie. Participants were asked to earn as many points as they could. One experimenter recorded the score after each round of the game (i.e., trial) and informed the participants about the total score at the end of each condition.

In the training phase, the participants practiced the task for the upcoming condition until they were acquainted with the rules. Before and after each experimental condition, there was 1 min of resting-state measurement. During the resting-state measurement, participants were asked to sit still with their eyes closed, relax their minds, and remain as motionless as possible. The reason to include a resting-state condition in the study was to have an additional control condition in which participants by no means interacted with each other.

**Figure 1:**
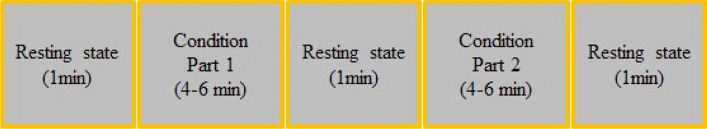
The structure of an experimental condition. All experimental conditions consisted of two parts with three resting-state phases before, in between, and after the condition.

As illustrated in Fig. 1, each condition consisted of two blocks of 30 trials divided by another 1 min of resting-state period. There were 60 trials in total in each condition, which were presented in a fixed order. The entire experiment lasted approximately 60 min together with the training phases. The entire lab visit took approximately 150 min depending on the preparation time.

**Figure 2:**
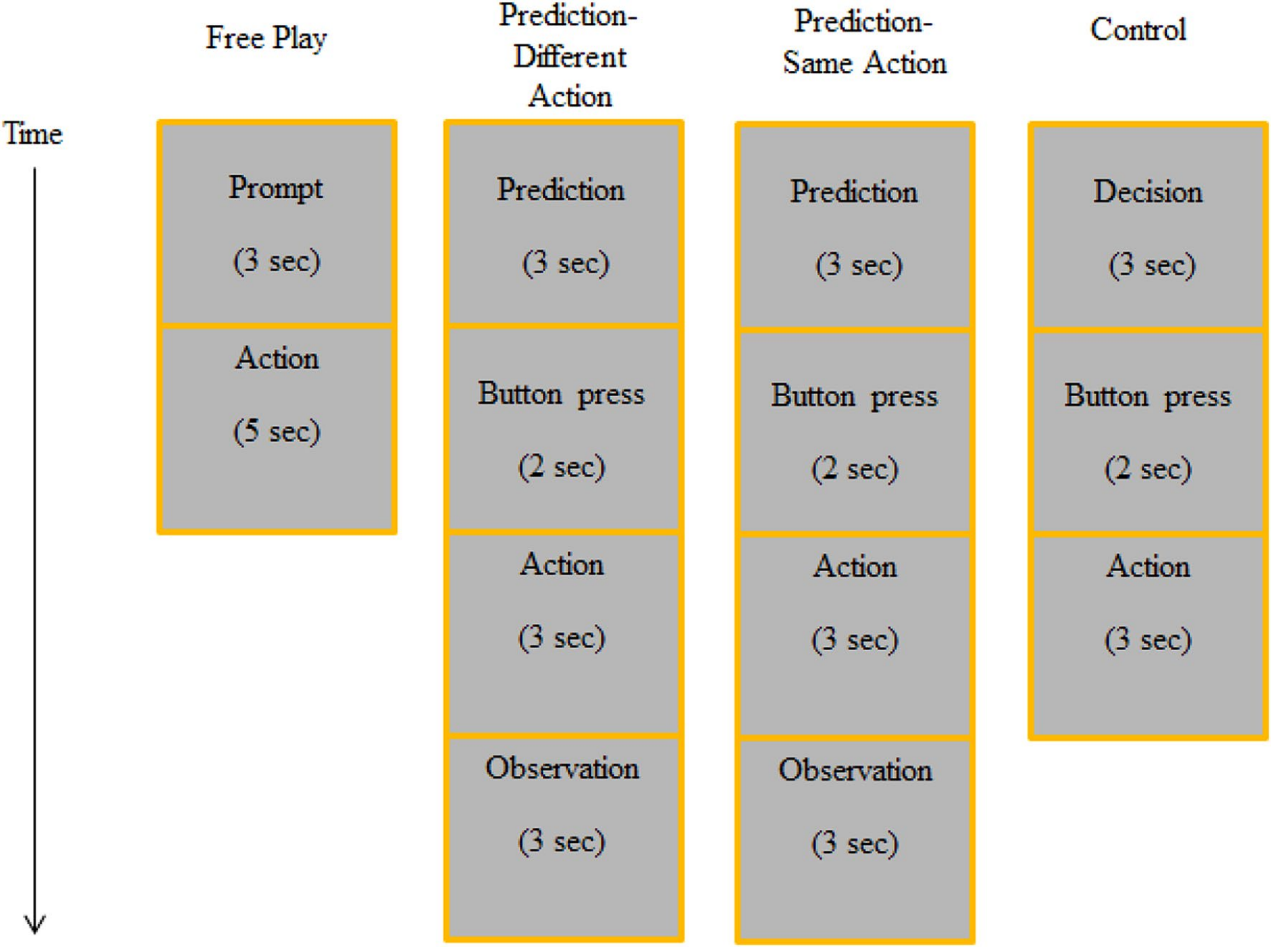
The structure and duration of four experimental conditions in the experiment.

As seen in Fig. 2, the experiment consisted of four conditions, each of which was performed once. Each session started with the *Free-Play* condition to avoid carry-over effects that might stem from explicit experimental manipulations in the other conditions. In the remaining conditions the order was randomized across dyads. Participants received written instructions about the tasks before each condition. In the *Free Play* condition, participants played the game as they would do in real-life without any explicit experimental manipulation. After participants heard the prompt “One, two, three”, they performed one of three gestures they chose for that particular trial (i.e., rock, paper, or scissors). Immediately after the participants performed the gestures they chose for that trial, the outcome of the game was evident to the players. In this condition, the participants might have made implicit predictions about the other player’s actions or not.

In the *Prediction-Different Action* condition, participants were instructed to explicitly predict which action the other player will perform in this particular trial (i.e., prediction phase, 3 s). After this phase, they were asked to press the button using their left index finger that corresponds to their prediction (i.e., button press phase, 2 s). Next, they performed the action using their right hand that would win against the predicted action (i.e., action phase, 3 s). Except for the *Free Play* condition, a panel was placed in between participants occluding the area around lower arms and the hands. After the action phase, the panel in between the participants was lowered so that the participants could see the hand gesture the other player performed in that round (i.e., observation phase, 3 s). This phase also revealed which participant won the game in that trial. Here, one of the participants earned points when they correctly guessed the action the other player would perform and made the action that would win against the other player’s action (e.g. prediction rock, action paper). Thus, this condition represented a competitive situation.

In the *Prediction-Same Action* condition, the game rules changed. Here, both participants won when they predicted and performed the same action as the other player (e.g. prediction rock, action rock). Therefore, a collaborative performance was generated. The structure of the trial was the same as in the *Prediction-Different Action* condition.

In the *Control* condition, each participant first had to decide on which action they will perform in that trial regardless of the other participant’s response (i.e., decision phase, 3 s). Then, they pressed the button indicating the gesture they decided to perform in this particular trial (i.e., button press, 2 s) following which they performed the gesture using their right hand (i.e., action phase, 3 s). No observation phase was included and no points were earned in this condition.

We opted for this trial structure because we concurrently measured fNIRS and electroencephalography (EEG) in this study. The reason why we measured both dual EEG and dual fNIRS was to double check the validity of the synchronization measures. Given that both measures worked successfully, we decided to report the result of the dual EEG analyses in a separate paper. We believe the vastness and complexity of the dual EEG data including synchronization measurements in different phases in each of the four conditions (together with the dual fNIRS results) would be a lot of information for a single paper, which might make it too difficult to follow for the reader. For the fNIRS analysis reported in this paper, we calculated the neural coherence for the entire duration of a trial combining all phases due to slower sampling rate and the slower emergence of the hemodynamic response captured with fNIRS as compared to EEG.

### Procedure

**Figure 3:**
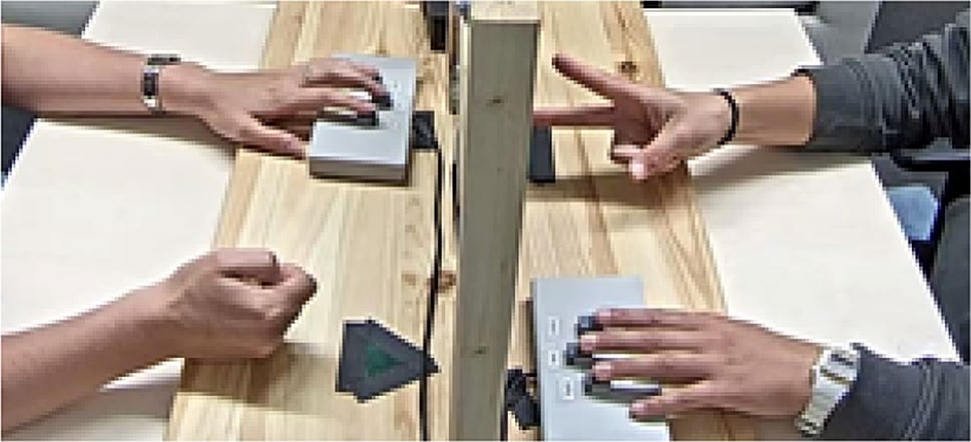
A picture of the experimental set-up. The participants sat on the left and the right side of the table. The experimenter operating the panel was located in the middle. Participants placed their left hand on the button press apparatus and performed the actions with their right hands without lifting their arms (note: Informed consent was obtained to use the images).

As shown in Fig. 3, participants sat around a table facing each other. Participants were asked to sit as motionless as possible. Each participant placed their left hand on a button press apparatus and their right hand at the designated spot on the table, palms facing down. The panel in between the participants was operated by the experimenter sitting in the middle. With a custom made Arduino set-up, the panel in between the participants went down when the experimenter pushed a button, and triggers marking this event were sent to the recording software. Two cameras were placed at opposite positions facing each participant. Another camera was located next to both participants to record the actions of both participants simultaneously.

### fNIRS recordings

**Figure 4:**
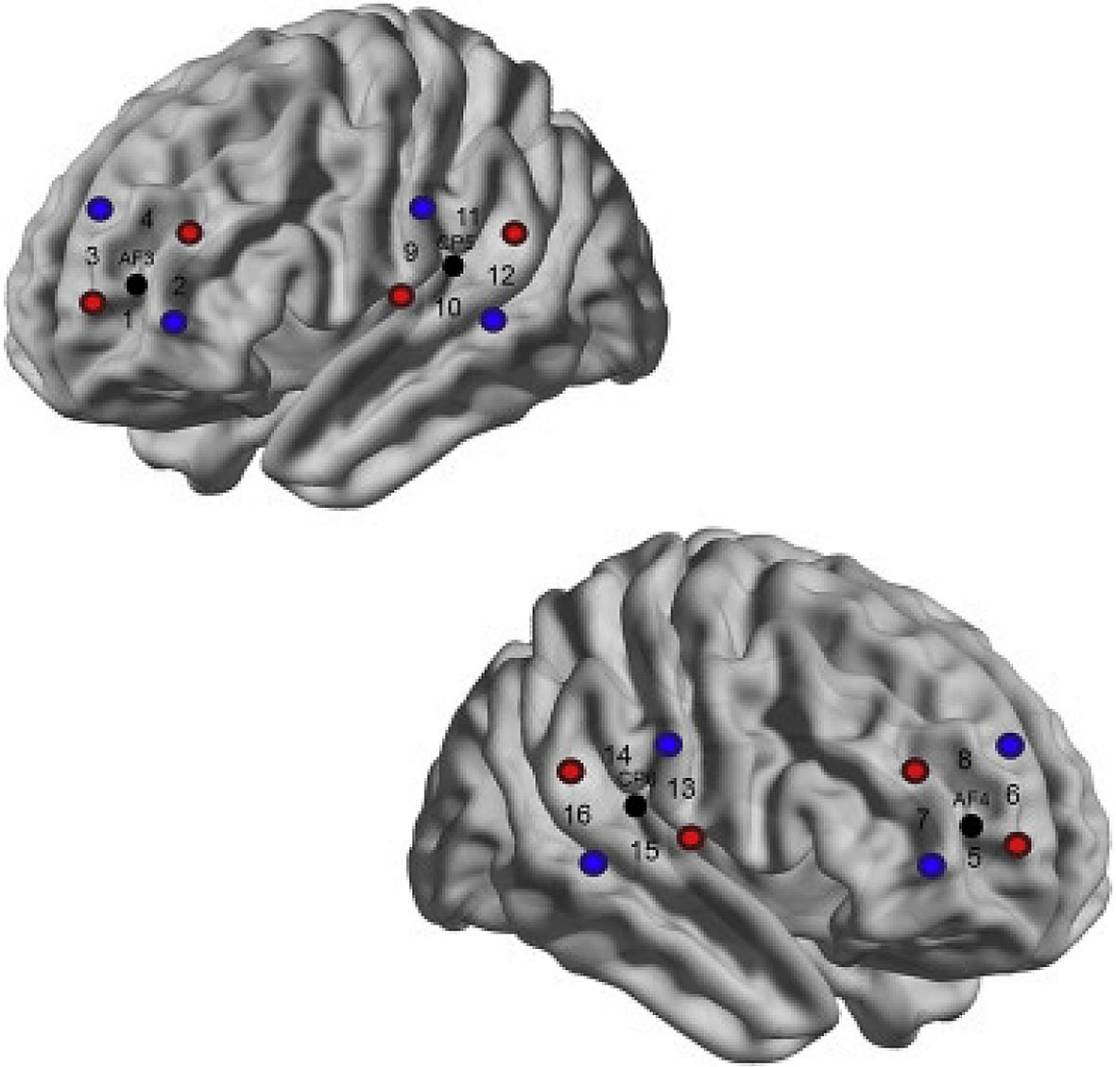
Cap configuration. Red circles illustrate sources and blue circles mark detectors. Channels between sources and detectors are marked by numbers 1–16. Black circles represent EEG 10–20 channel positions for orientation (Adapted from Nguyen et al. 2020a with permission).

We used a NIRScout 16–16 (NIRx Medizintechnik GmbH, Germany) Optical Topography system to record oxy-hemoglobin (HbO) and deoxy-hemoglobin (HbR) concentration changes. We used the same EEG cap with a 10–20 layout to place the four 2 × 2 fNIRS probe sets and the EEG electrodes. As illustrated in Fig. 4, in each probe set, 8 sources and 8 detectors were positioned, which resulted in 16 measurement channels with equal distances of 3 cm between the optodes. Based on standard electrode locations, we placed two probe sets over the left and right DLPFC surrounding F3 and F4. The remaining two probe sets were located on the left and right TPJ neighboring CP5 and CP6. The regions of interest were chosen based on the previous hyperscanning literature on social mentalizing^8,11,19,22^. The absorption of near-infrared light was measured at the wavelengths of 760 and 850 mm and the sampling frequency was 7.81 Hz.

### fNIRS processing

First, we visually inspected the data of each participant and performed a data quality check. We removed all channels in which a clear heart band was not observed. Accordingly, 83% of the channels from the whole sample were included in the final analysis. We then checked whether each participant had at least two good channels per region of interest (i.e., TPJ and DLPFC). This was the case for all participants. As the next step, we preprocessed the data using functions of Homer 2 operated in MATLAB. We corrected raw optical density data for motion with a smoothing procedure based on local regression using weighted linear least squares and a second-degree polynomial model^28^. We have used the function “hmrMotionCorrectSpline” with *p* = 0.99 (as suggested by default). We chose this method, as it yields a significant reduction in mean square error in the recovery of the HRF^29^. We then applied a bandpass filter with a high and low cut-off at 0.50 and 0.01 Hz^11,30^. Based on the modified Beer–Lambert Law, we then converted the filtered data to HbO and HbR values.

We used WTC to assess the relation between two fNIRS time series in each dyad and each channel as an index of neural synchrony^31,32^. WTC was calculated using the “Morlet” wavelet, three cycles per frequency and 12 sub-octaves per octave using the function wtc from the Cross Wavelet and Wavelet Coherence Toolbox^32^.We used the WTC function to calculate coherence across the entire condition, but not on a trial-by-trial basis, given the slow trajectory of hemodynamic response. Accordingly, coherence was estimated for at least 2 min of recording in each part of the condition (separated by a resting state phase). These values were subsequently averaged. Thus, the coherence values for each condition were based on at least 4 min long HbO and HbR time-series. The resting phase coherence was calculated and averaged over all 1 min long resting phases, before, in between and after each part of the condition. Based on previous literature (e.g.^22^) and spectral analyses, we examined 0.05–0.16 Hz as the frequency band of interest. The cycle size for the frequencies in the frequency band of interest ranged from 6 to 20 s comprising both trial durations of 8 s (*Free Play* and *Control* conditions) and 15 s (prediction conditions). In other words, the frequency band of interest incorporated all trial structures of the four different conditions. We then calculated coherence values in each channel for the four experimental conditions as well as the average of 12 resting-state phases to obtain values for a single resting-state condition. For each dyad, we obtained 5 (conditions) × 16 (channels) coherence values. The codes used in the analysis of the current study can be accessed at this link: https://github.com/tnguyen1992/RPS. A figure further depicting how coherence values were calculated can be found in Supplementary Materials. For further resources on a similar approach regarding fNIRS processing, please see https://osf.io/wspz4/.

### Statistical approach

Statistical analyses were run in RStudio (Rstudio Team, 2015) using the glmmTMB package^33^ for generalized linear mixed-effects modeling (GLMM). WTC values were entered as the response variable. Because all values ranged from 0 to 1, we set the model to assume a beta distribution. Condition and region of interest were then added as fixed effects as well as an interaction effect. A full random effects structure was built including random slopes for the fixed and interaction effects in random intercepts for each dyad. Random slopes were subsequently removed to reach convergence for each model. We visually checked the distribution of the fitted residuals for a normal distribution. Next, we used the emmeans/emtrends function from the emmeans package^34^ to run post-hoc analyses. We corrected *p*-values for multiple comparisons during post-hoc contrasts of factorial fixed and interaction effects with more than two levels using Tukey’s Honest Significant Difference^35^.

We conducted random pair analyses to test for the significance of neural synchrony values above spurious correlations that occur within the signal. We used a permutation approach, by pairing Subject 1 with 1000 random Subject 2 and subsequently averaged the coherence value for each Subject 1 in each channel and condition. Accordingly, each dyad’s coherence value (Subject 1 and original partner Subject 2) was compared to a randomized dyad’s value (Subject 1 and random partners Subject 2).

Overall, the following two models were tested (* indicates the inclusion of both fixed and interaction effects):

1. Control Analysis HbO/HbR
  WTC ~ random pair * condition * region + (1+random pair*condition * region | dyad)
2. Main Analysis HbR
  WTC ~ condition * region + (1+ condition * region | dyad)

## Results

### Control analyses

Firstly, we conducted control analyses to compare neural coherences in original dyads against neural coherence in randomly paired dyads. The findings for the control analysis in HbO values showed that generally there was no difference between coherence values of original and random pairs, *p* = 0.550. Even though there were significant main effects for condition, χ^2^ (3) = 31.06, *p* < 0.001, region, χ^2^ (3) = 24.60, *p* < 0.001 as well as an interaction effect between region and original vs. random pairs, χ^2^ (3) = 9.27, *p* = 0.025, we found no significant increase in coherence for original pairs in interaction with the condition or with certain regions in certain conditions, *p* > 0.54.

To further elucidate the differences between original and random pairs, we conducted post-hoc contrasts for pairings in each condition and each region of interest. For all of the post-hoc analyses, we corrected the *p* values for multiple comparisons using Tukey’s Honest Significant Difference. The contrasts revealed a marginally higher coherence value for original dyads during the *Free Play* condition in the left DLPFC, *t* = 1.92, *p* = 0.055, while all other contrasts showed no significant differences between original and random pairs, *p* > 0.17.

**Figure 5:**
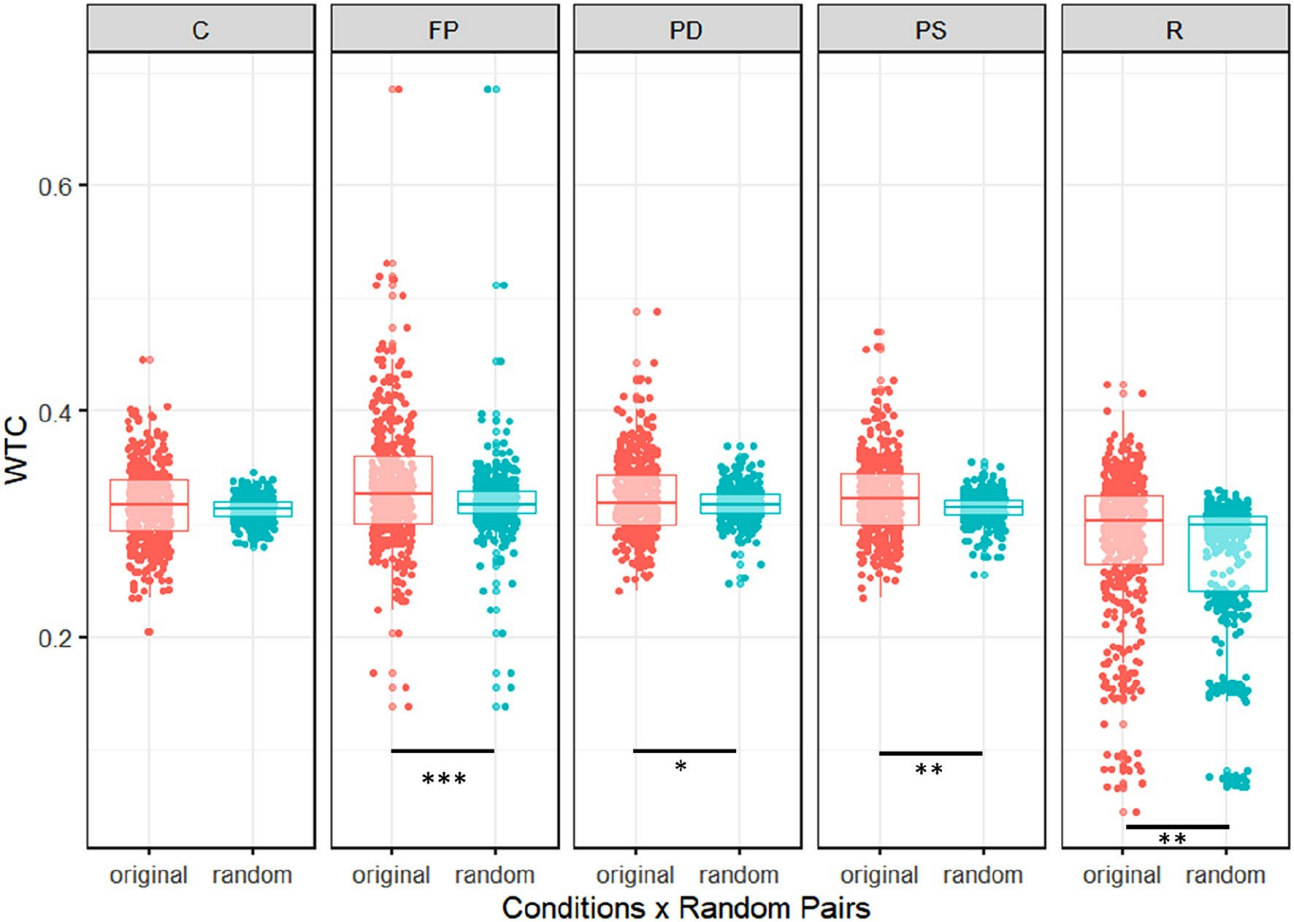
Box plots illustrating WTC values for each experimental condition for original and random pairs for HbR. *Free Play* (FP), *Prediction-Different Action* (PD) and *Prediction-Same Action* (PS) conditions and an average of all resting state phases (R) showed higher coherence in original pairs than in random pairs. The *Control* (C) condition revealed no significant differences between original and random pairs. *p ≤ 0.05, **p ≤ 0.01, ***p ≤ 0.001.

Next, we estimated the same linear mixed model for HbR values. Here, we found a significant main effect of original vs. random pairs, χ^2^ (1) = 8.84, *p* = 0.003 as well as an interaction effect between original vs. random pairs and condition, χ^2^ (4) = 17.45, *p* = 0.002 (see Fig. 5). Furthermore, the main effect of condition, χ^2^ (4) = 29.51, *p* < 0.001, as well as region, χ^2^ (3) = 24.41, *p* < 0.001, were significant. All further interaction effects, namely pairing and region, condition, and region as well as pairing, condition, and region, remained non-significant, *p* > 0.083.

We calculated post-hoc contrasts and found increased coherence for original dyads during the *Free Play* condition in the left DLPFC, *t* = 2.07, *p* = 0.038, as well as the right TPJ, *t* = 3.22, *p* = 0.001. Furthermore, data revealed increased coherence for original dyads in the *Prediction-Different Action* condition in the left TPJ, *t* = 2.24, *p* = 0.024. Original dyads also showed increased coherence in the *Prediction-Same Action* condition in the left dlPFC, *t* = 2.64, *p* = 0.008. All other contrasts were either marginal or non-significant, all *p*s > 0.055. When the contrast between original and random pairs was averaged over all regions, all task conditions (i.e. *Free Play, Prediction- Different, Prediction-Same*) as well as resting phases, analyses revealed higher coherence values for original than random pairs, *t* > 2.11, *p* < 0.034 (see Fig. 5). Only the *Control condition* did not reveal differences between original and random pairs, *p* > 0.490. Model output for the control analysis can be found in Supplementary Material Table S1.

Due to the fact that control analyses revealed significant synchronization in HbR values, but no significant neural synchronization between participants in HbO values, we continued our main analyses with HbR values. This approach is consistent with studies showing that HbR values are less prone to artifacts arising from extracerebral processes, thus providing more robust results^36,37^.

### Main analyses

To examine whether participants showed increased neural synchrony during a collaborative vs. competitive prediction game, we compared the *Free Play* condition to *Prediction-Different Action* condition as well as *Prediction-Same Action* condition, with the latter two having explicit instructions to predict the other player’s actions. Moreover, neural synchrony values in all experimental conditions were contrasted with two control conditions that included no interaction and explicit response. The two control conditions were as follows: (1) a resting-state condition averaged across all resting state conditions (2) the *Control* condition, in which participants played on their own. For all of the post-hoc analyses, we corrected the *p* values for multiple comparisons using Tukey’s Honest Significant Difference.

**Figure 6:**
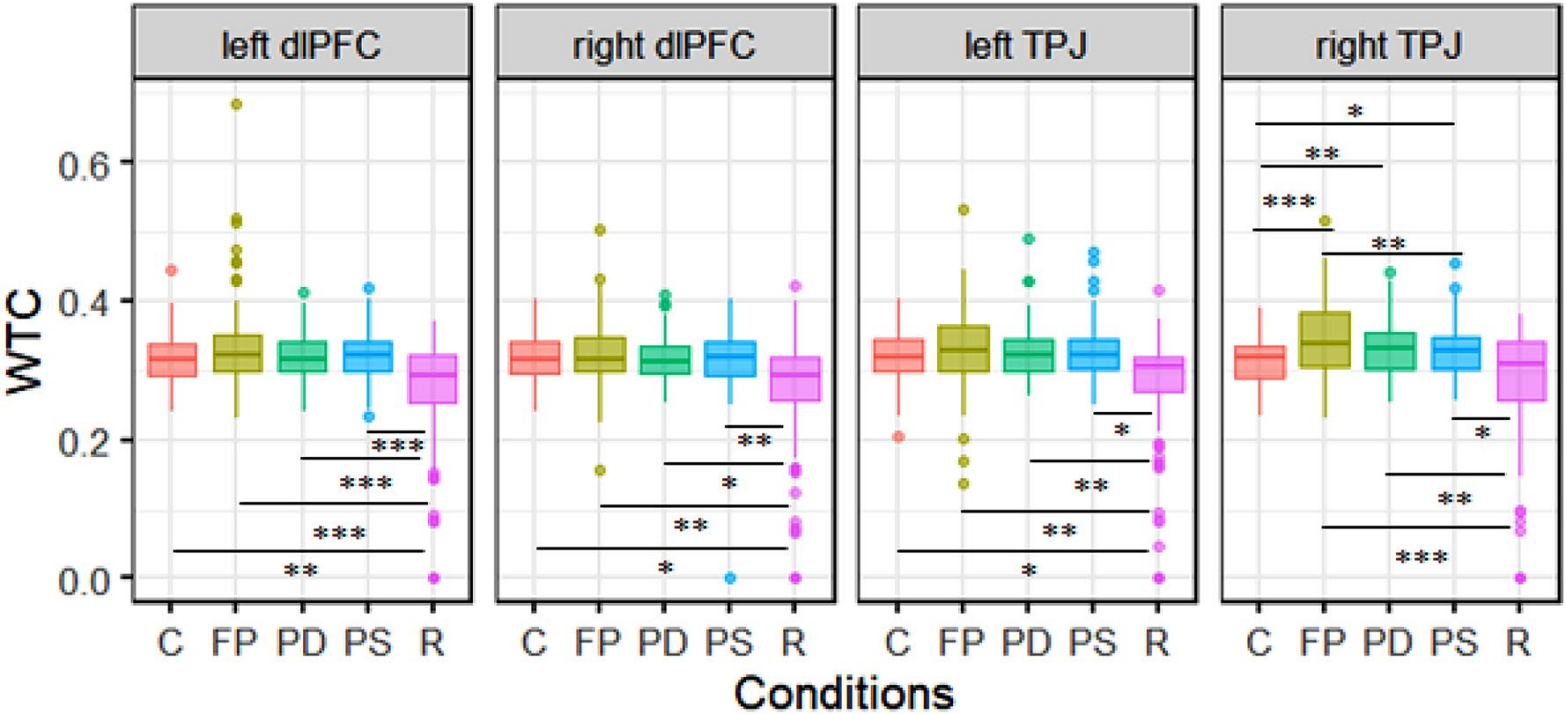
Box plots illustrating WTC values for each experimental condition for each region separately. In the right TPJ, *Free Play* (FP), *Prediction-Different* (PD) and *Prediction-Same* (PS) conditions showed higher coherence than during the *Control* (C) condition, *p* < .023 and neural synchrony was significantly higher in *FP* condition as compared to the *PS* condition, *p* = .010. Neural synchrony in *FP* condition was also marginally higher than in *C* condition in the left DLPFC, *p* = .057. *p ≤ 0.05, **p ≤ 0.01, ***p ≤ 0.001.

**Figure 7:**
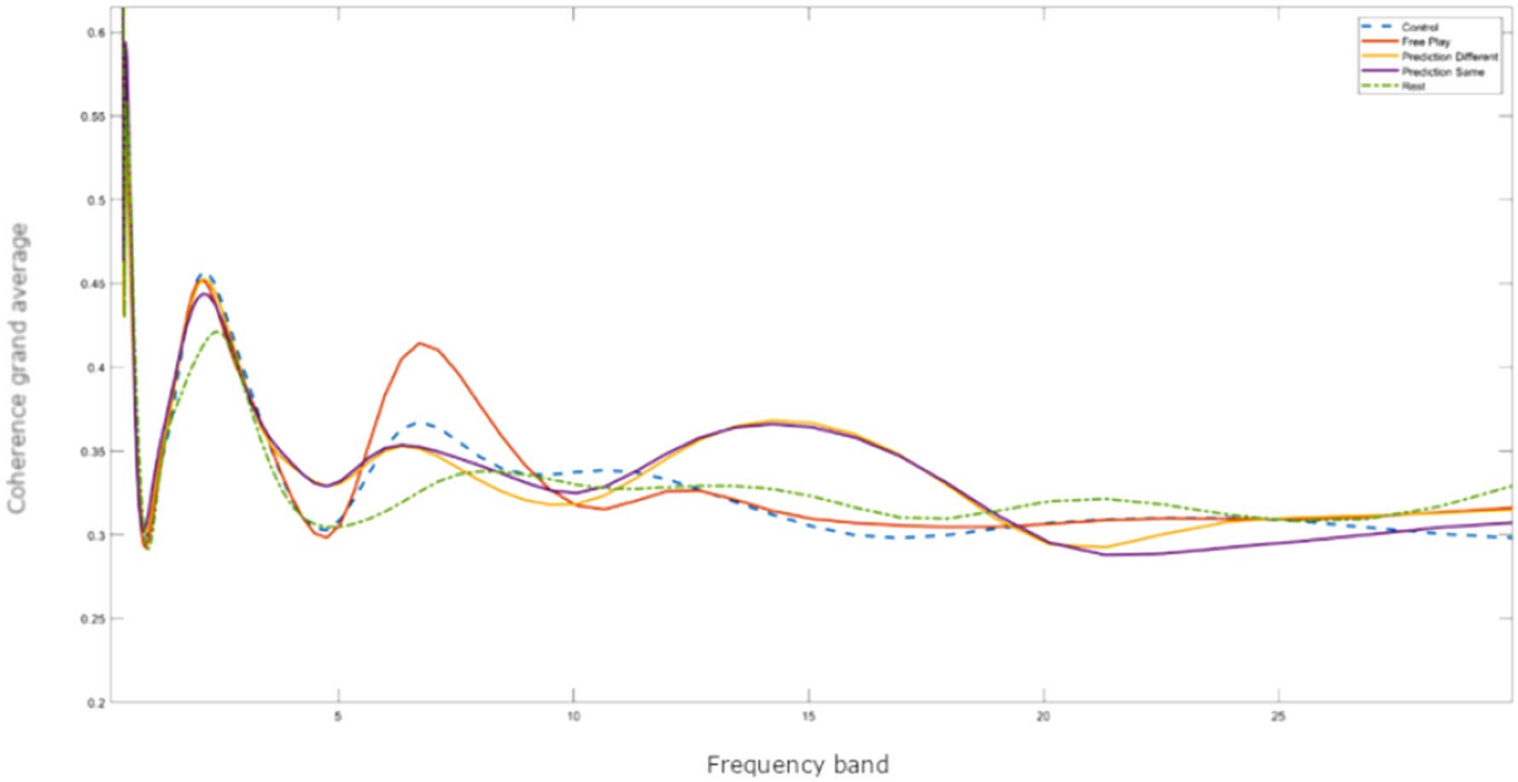
A plot displaying the grand average of coherence over power in all of the experimental conditions and the resting state. The coherence grand average (y axis) over the whole length of the condition frequency band (x axis) in the different conditions: *Control* (blue), *Free Play* (red), *Prediction-Different Action* (yellow), *Prediction-Same Action* (magenta), Rest (green).

The results from Model 2 depict a main effect of condition, χ^2^(4) = 22.30, *p* < 0.001, a main effect of region, χ^2^(3) = 31.95, *p* = 0.001 and an interaction effect between condition and region, χ^2^(12) = 25.22, *p* = 0.010. As illustrated in Fig. 6, post-hoc contrasts revealed that in all regions neural synchronization was higher in experimental conditions than in the resting state condition, all *ps* < 0.05, except for the right TPJ, *p* = 0.213. In the right TPJ, all interaction conditions (i.e., *Free Play, Prediction-Same, Prediction-Different*) showed higher coherence than during the *Control* condition, *t* > 2.98, *p* < 0.023. In addition, neural synchrony was significantly higher in the *Free Play* condition as compared to the *Prediction-Same Action* condition, *t* = 3.25, *p* = 0.010. In Fig. 7, we include a plot displaying the grand average of coherence over power in all conditions and rest. Model output for the main analysis and model contrasts can be found in Supplementary Material Table S2 and S3.

We further analyzed whether (a) concurrent movement, assessed by reaction time differences to the button press in the control and both explicit prediction conditions, (b) mutual correct prediction of the same action (i.e., prediction accuracy) influenced neural synchrony. The models displayed no significant relations of aforementioned variables to neural synchrony, *p* > 0.201. More information on these analysis can be found in the Supplementary Materials.

## Discussion

We examined whether there is increased neural synchrony when predicting others’ actions. Participants played variations of a Rock-Paper-Scissors game while we measured their neural coherence using dual fNIRS. In all regions of interest (i.e., left and right DLPFC and the TPJ) we observed higher neural coherence in experimental conditions as compared to a resting-state condition averaged across all resting state conditions. Importantly, in the right TPJ, neural coherence was higher in all of the interaction conditions (i.e., *Free Play, Prediction-Same Action, Prediction-Different Action*) as compared to a *Control* condition in which participants played the game on their own. Unexpectedly, we observed the highest neural synchronization in the right TPJ in the *Free Play* condition in which participants played the game as they would do in real life where they might have made implicit predictions about the other player’s actions. In contrast to our hypotheses, we found no evidence of increased neural synchrony when participants made explicit predictions about others’ actions in the *Prediction-Same Action* and *Prediction-Different Action* conditions.

Several studies have stressed the role of the right TPJ in social interactions, particularly when participants were asked to mentalize or take the perspective of other social agents^25,38,39^. In line with the previous literature, we observed higher neural coherence in conditions in which participants engaged in a non-verbal interaction with each other such as playing a game as compared to when there was no interaction between the participants (e.g. in the *Control* condition). This finding serves as a proof-of-concept, as it shows that neural coherence observed in our experiment is not merely an epiphenomenon. In other words, because we observed higher neural coherence only when participants engaged in a dynamic interaction, it is unlikely that enhanced activation observed in right TPJ regions occurs simply because participants received the same environmental input^40^.

In contrast to our hypotheses, however, we observed no evidence of enhanced neural coherence for conditions in which participants were asked to explicitly predict each other’s actions. It could be that our hypotheses were not correct or our experimental manipulation did not work. That is, participants chose a random action in all conditions and did not make an educated guess about the other player’s actions in prediction conditions. Alternatively, it could be that participants tried to make an educated guess about the other player’s move in all of the interaction conditions, regardless of explicit instructions. This implies that participants implicitly predicted the other player’s actions in the *Free Play* condition. Although applying a prediction strategy in each game might be a possibility, it is unclear why implicit predictions would lead to significantly higher coherence values in the *Free Play* condition as compared to, for example, the *Prediction-Same Action* condition in which participants were asked to collaborate.

One could argue that the prediction conditions were too demanding for the participants, as they might have disrupted the natural interaction between participants. Particularly, the *Prediction-Same Action* condition might have posed an additional challenge to participants, as they were expected to overwrite the original game rules in order to perform the same action to win points. The competitive nature of the original game might have made it challenging for the participants to perform the game in a collaborative fashion, which might have resulted in the lack of high coherence in this condition in contrast to our hypotheses. Although participants went through a training phase before each condition to ensure that they understood the task requirements, some might have struggled to maintain optimal performance throughout the condition. However, we find this explanation rather unlikely for two reasons: (1) As reported in the Supplementary Materials, we did not find any effect of reaction time differences on neural coherence values across conditions. (2) Studies show that, on the contrary, participants tend to synchronize their motor behavior and imitate each other’s actions, even when this outcome is not desirable for optimal performance^2,7^. Therefore, it is reasonable to assume that task demands in the *Prediction-Same Action* condition were manageable for the participants.

One explanation for observing the highest neural coherence in a free play situation as compared to the explicit prediction conditions could be that the explicit prediction conditions were highly structured, which might have hindered the participants to fully engage in a naturalistic interaction. It is conceivable that they were focused on their own performance as well as monitoring the other’s performance, which might have prevented them from naturally interacting with each other in the *Prediction-Same* and *Prediction-Different* conditions. This supports the key idea that neural coherence emerges when individuals engage in a dynamic mutual real-life interaction^11,19^. Moreover, these findings are in line with recent theoretical arguments on interpersonal synchrony suggesting that it occurs even in the absence of a common goal^1^. In the current study, participants shared a common goal only in the *Prediction-Same* condition; however, this condition did not elicit the highest neural synchrony values. Taken together, this finding suggests that it is the natural interaction itself that modulate dynamic mutual alignment of behavior and brain responses regardless of a common goal.

It should be noted that all the results discussed here are based on HbR changes. Most prior fNIRS-based hyperscanning studies (e.g.^10,12,30^) was only based on HbO values due to the higher signal-to-noise ratio as well as increased sensitivity to changes in the regional cerebral blood flow in single fNIRS measurements^41^. However, it is unclear whether HbO or HbR is more relevant to fNIRS-based hyperscanning. Recently, an increasing number of studies have presented results based on both HbO and HbR or only on HbR changes (e.g.^42–45^), due to the greater spatial specificity of the HbR^46^ and its higher correlation with the blood oxygen level dependent (BOLD) signal acquired during fMRI^47^. These studies revealed different patterns of neural synchronization between HbO- and HbR-based signals. Similarly, we also found a significant difference between conditions based on HbR signals only. Therefore, we think that it would be informative to examine and report interpersonal neural synchronization based on both HbO and HbR signals in future fNIRS-based hyperescanning studies.

Using temporally sensitive measures such as EEG are needed to clarify the role of prediction for interpersonal synchrony. One could argue that the fNIRS method was not sensitive enough to reveal the examined effects. In other words, the process of predicting another person’s actions might indeed enhance interpersonal synchrony; however, capturing this effect requires a more fine-tuned approach with high temporal precision. As mentioned above, in this study, we included the whole trial sequence in the analysis, not only the prediction phase, because of the timing characteristics of the hemodynamic response captured by the fNIRS. Results of our dual-EEG study will be highly informative to further examine the link between explicit predictions and interpersonal synchrony.

To summarize, in this paper, we investigated whether there is enhanced neural synchronization when individuals explicitly predict others’ actions. Participants performed variations of a Rock-Paper-Scissors game while we measured the dynamic alignment of their brain activity using dual-fNIRS. We found increased neural coherence in the right TPJ when participants played the game naturally as they would do in real life as compared to when they played the game on their own. In contrast to our hypotheses, we found no evidence of enhanced neural synchrony when participants explicitly predicted others’ actions. These findings suggest that neural coherence emerges during dynamic mutual interaction regardless of a common goal or an explicit prediction strategy. In future studies, we aim to shed light on the fine-tuned temporal dynamics of interpersonal synchrony and predictions using dual EEG.

## Supplementary Information

Supplementary Information.

## Data Availability

The datasets generated during and/or analyzed during the current study are available from the corresponding author on reasonable request.

## References

[1] Gallotti M, Fairhurst MT, Frith CD (2017). Alignment in social interactions. Conscious. Cognit..

[2] Cook R, Bird G, Lünser G, Huck S, Heyes C (2012). Automatic imitation in a strategic context: Players of rock–paper–scissors imitate opponents' gestures. Proc. R. Soc. B Biol. Sci..

[3] Koban L, Ramamoorthy A, Konvalinka I (2019). Why do we fall into sync with others? Interpersonal synchronization and the brain's optimization principle. Soc. Neurosci..

[4] Shamay-Tsoory SG, Saporta N, Marton-Alper IZ, Gvirts HZ (2019). Herding brains: A core neural mechanism for social alignment. Trends Cognit. Sci..

[5] Cho PS, Escoffier N, Mao Y, Green C, Davis RC (2020). Beyond physical entrainment: Competitive and cooperative mental stances during identical joint-action tasks differently affect inter-subjective neural synchrony and judgements of agency. Soc. Neurosci..

[6] Hill CA (2017). A causal account of the brain network computations underlying strategic social behavior. Nat. Neurosci..

[7] Dumas G, Nadel J, Soussignan R, Martinerie J, Garnero L (2010). Inter-brain synchronization during social interaction. PLoS ONE.

[8] Liu T, Saito G, Lin C, Saito H (2017). Inter-brain network underlying turn-based cooperation and competition: A hyperscanning study using near-infrared spectroscopy. Sci. Rep..

[9] Sun H (2019). Framing a trust game as a power game greatly affects interbrain synchronicity between trustor and trustee. Soc. Neurosci..

[10] Cui X, Bryant DM, Reiss AL (2012). NIRS-based hyperscanning reveals increased interpersonal coherence in superior frontal cortex during cooperation. Neuroimage.

[11] Nguyen T (2020). The effects of interaction quality on neural synchrony during mother-child problem solving. Cortex.

[12] Reindl V, Gerloff C, Scharke W, Konrad K (2018). Brain-to-brain synchrony in parent-child dyads and the relationship with emotion regulation revealed by fNIRS-based hyperscanning. NeuroImage.

[13] Sinha, N. *et al.* EEG hyperscanning study of inter-brain synchrony during cooperative and competitive interaction. In *2016 IEEE International Conference on Systems, Man, and Cybernetics (SMC)* IEEE , 004813–004818. 10.1109/SMC.2016.7844990 (2016).

[14] Friston KJ, Frith CD (2015). A Duet for one. Conscious. Cognit..

[15] Friston KJ, Frith CD (2015). Active inference, communication and hermeneutics. Cortex.

[16] Schoot L, Hagoort P, Segaert K (2016). What can we learn from a two-brain approach to verbal interaction?. Neurosci. Biobehav. Rev..

[17] Jiang J, Zheng L, Lu C (2021). A hierarchical model for interpersonal verbal communication. Soc. Cognit. Affect. Neurosci..

[18] Hasson U, Frith CD (2016). Mirroring and beyond: Coupled dynamics as a generalized framework for modelling social interactions. Philos. Trans. R. Soc. B Biol. Sci..

[19] Nguyen T (2020). Neural synchrony in mother-child conversation: Exploring the role of conversation patterns. Soc. Cognit. Affect. Neurosci..

[20] Hamilton AFD (2021). Hyperscanning: Beyond the hype. Neuron..

[21] Hoehl S, Fairhurst M, Schirmer A (2021). Interactional synchrony: Signals, mechanisms and benefits. Soc. Cognit. Affect. Neurosci..

[22] Jiang J (2012). Neural synchronization during face-to-face communication. J. Neurosci..

[23] Koster-Hale J, Saxe R (2013). Theory of mind: A neural prediction problem. Neuron.

[24] van Pelt S (2016). Beta-and gamma-band activity reflect predictive coding in the processing of causal events. Soc. Cognit. Affect. Neurosci..

[25] Wang Z, Wang Y, Zhou X, Yu R (2020). Interpersonal brain synchronization under bluffing in strategic games. Soc. Cognit. Affect. Neurosci..

[26] Li R, Mayseless N, Balters S, Reiss AL (2021). Dynamic inter-brain synchrony in real-life inter-personal cooperation: A functional near-infrared spectroscopy hyperscanning study. Neuroimage.

[27] Czeszumski A (2021). Cooperative behavior evokes inter-brain synchrony in the prefrontal and temporoparietal cortex: A systematic review and meta-analysis of fNIRS hyperscanning studies. bioRxiv..

[28] Scholkmann F, Spichtig S, Muehlemann T, Wolf M (2010). How to detect and reduce movement artifacts in near-infrared imaging using moving standard deviation and spline interpolation. Physiol. Meas..

[29] Cooper R (2012). A systematic comparison of motion artifact correction techniques for functional near-infrared spectroscopy. Front. Neurosci..

[30] Baker JM (2016). Sex differences in neural and behavioral signatures of cooperation revealed by fNIRS hyperscanning. Sci. Rep..

[31] Chang C, Glover GH (2010). Time–frequency dynamics of resting-state brain connectivity measured with fMRI. Neuroimage.

[32] Grinsted A, Moore JC, Jevrejeva S (2004). Application of the cross wavelet transform and wavelet coherence to geophysical time series. Nonlinear Process. Geophys..

[33] Brooks ME (2017). glmmTMB balances speed and flexibility among packages for zero-inflated generalized linear mixed modeling. R J..

[34] Lenth, R. Emmeans: Estimated marginal means, aka least-squares means. R package version 1.4.2. Available at: https://CRAN.R-project.org/package=emmeans (2019).

[35] Abdi, H., & Williams, L. J. Tukey’s honestly significant difference (HSD) test*.* In *Encyclopedia of Research Design*, 6 SAGE Publications, Inc (2010).

[36] Tachtsidis I, Scholkmann F (2016). False positives and false negatives in functional near-infrared spectroscopy: Issues, challenges, and the way forward. Neurophotonics.

[37] Piazza EA, Hasenfratz L, Hasson U, Lew-Williams C (2020). Infant and adult brains are coupled to the dynamics of natural communication. Psychol. Sci..

[38] Lombardo MV (2011). Specialization of right temporo-parietal junction for mentalizing and its relation to social impairments in autism. Neuroimage.

[39] Saxe R, Wexler A (2005). Making sense of another mind: The role of the right temporo-parietal junction. Neuropsychologia.

[40] Fishburn FA (2018). Putting our heads together: Interpersonal neural synchronization as a biological mechanism for shared intentionality. Soc. Cognit. Affect. Neurosci..

[41] Hoshi Y (2003). Functional near-infrared optical imaging: Utility and limitations in human brain mapping. Psychophysiology.

[42] Reindl V (2022). Multimodal hyperscanning reveals that synchrony of body and mind are distinct in mother-child dyads. Neuroimage.

[43] Cañigueral R (2021). Facial and neural mechanisms during interactive disclosure of biographical information. Neuroimage.

[44] Rojiani R, Zhang X, Noah A, Hirsch J (2018). Communication of emotion via drumming: Dual-brain imaging with functional near-infrared spectroscopy. Soc. Cognit. Affect. Neurosci..

[45] Noah JA (2020). Real-time eye-to-eye contact is associated with cross-brain neural coupling in angular gyrus. Front. Hum. Neurosci..

[46] Dravida S, Noah JA, Zhang X, Hirsch J (2017). Comparison of oxyhemoglobin and deoxyhemoglobin signal reliability with and without global mean removal for digit manipulation motor tasks. Neurophotonics.

[47] Sato H (2013). A NIRS–fMRI investigation of prefrontal cortex activity during a working memory task. Neuroimage.

