# Supplementary material for: Interpersonal neural synchrony when predicting others’ actions during a game of rock-paper-scissors
Source: Sci Rep. 2022 Jul 28;12:12967. doi: 10.1038/s41598-022-16956-z (PMC9334613; doi:10.1038/s41598-022-16956-z)
Supplement: Supplementary file 1 — Supplementary Information. [file 41598_2022_16956_MOESM1_ESM.docx]

**Supplementary Materials**

**Supplementary tables**

Table S1: Control Analysis Output for HbR values

|  | *Estimates* | *SE* | *CI Lower* | *CI Upper* | *X²* | *df* | *p* |
| --- | --- | --- | --- | --- | --- | --- | --- |
| Model 1: *wtc ~ pairing * condition * region* | | | | | | | |
| (Intercept) | 0.314 | 0.003 | 0.307 | 0.321 |  |  |  |
| pairing |  |  |  |  | 8.84 | 1 | .003 |
| random | -0.002 | 0.004 | -0.010 | 0.005 |  |  |  |
| condition |  |  |  |  | 29.52 | 4 | <.001 |
| condition (FP) | 0.024 | 0.008 | 0.008 | 0.039 |  |  |  |
| condition (PD) | 0.005 | 0.004 | -0.003 | 0.013 |  |  |  |
| condition (PS) | 0.007 | 0.004 | -0.002 | 0.015 |  |  |  |
| condition (R) | -0.047 | 0.014 | -0.076 | -0.019 |  |  |  |
| region |  |  |  |  | 24.41 | 3 | <.001 |
| region (right dlPFC) | 0.001 | 0.004 | -0.007 | 0.009 |  |  |  |
| region (left TPJ) | 0.006 | 0.004 | -0.003 | 0.014 |  |  |  |
| region (right TPJ) | -0.001 | 0.005 | -0.011 | 0.007 |  |  |  |
| pairing : condition |  |  |  |  | 17.45 | 4 | .002 |
| random : FP | -0.011 | 0.007 | -0.025 | 0.002 |  |  |  |
| random : PD | -0.003 | 0.005 | -0.014 | 0.006 |  |  |  |
| random : PS | -0.008 | 0.005 | -0.018 | 0.002 |  |  |  |
| random : R | -0.006 | 0.005 | -0.017 | 0.004 |  |  |  |
| pairing : region |  |  |  |  | 7.55 | 3 | .056 |
| random : right dlPFC | -0.001 | 0.005 | -0.011 | 0.009 |  |  |  |
| random : left TPJ | -0.005 | 0.005 | -0.016 | 0.004 |  |  |  |
| random : right TPJ | 0.004 | 0.005 | -0.006 | 0.015 |  |  |  |
| condition : region |  |  |  |  | 11.89 | 12 | .454 |
| pairing : condition : region |  |  |  |  | 12.04 | 12 | .443 |

Table S2: Main Analysis Output for HbR values

|  | *Estimates* | *SE* | *CI Lower* | *CI Upper* | *X²* | *df* | *p* |
| --- | --- | --- | --- | --- | --- | --- | --- |
| Model 2: *wtc ~ condition * region* | | | | | | | |
| (Intercept) | 0.314 | 0.004 | 0.307 | 0.321 |  |  |  |
| condition |  |  |  |  | 22.30 | 4 | <.001 |
| condition (FP) | 0.025 | 0.009 | 0.006 | 0.043 |  |  |  |
| condition (PD) | 0.003 | 0.005 | -0.005 | 0.012 |  |  |  |
| condition (PS) | 0.007 | 0.005 | -0.002 | 0.016 |  |  |  |
| condition (R) | -0.043 | 0.011 | -0.066 | -0.019 |  |  |  |
| region |  |  |  |  | 31.95 | 3 | .001 |
| region (right dlPFC) | 0.001 | 0.005 | -0.007 | 0.010 |  |  |  |
| region (left TPJ) | 0.005 | 0.004 | -0.003 | 0.014 |  |  |  |
| region (right TPJ) | -0.001 | 0.005 | -0.011 | 0.008 |  |  |  |
| condition : region |  |  |  |  | 25.22 | 12 | .010 |
| FP : right dlPFC | -0.020 | 0.009 | -0.039 | 0.000 |  |  |  |
| PD : right dlPFC | -0.004 | 0.007 | -0.017 | 0.008 |  |  |  |
| PS : right dlPFC | -0.003 | 0.006 | -0.016 | 0.009 |  |  |  |
| R : right dlPFC | 0.004 | 0.007 | -0.009 | 0.016 |  |  |  |
| FP : left TPJ | -0.017 | 0.012 | -0.042 | 0.007 |  |  |  |
| PD : left TPJ | 0.005 | 0.007 | -0.009 | 0.020 |  |  |  |
| PS : left TPJ | -0.001 | 0.006 | -0.014 | 0.012 |  |  |  |
| R : left TPJ | 0.005 | 0.006 | -0.006 | 0.016 |  |  |  |
| FP : right TPJ | 0.010 | 0.010 | -0.009 | 0.030 |  |  |  |
| PD : right TPJ | 0.016 | 0.007 | 0.001 | 0.030 |  |  |  |
| PS : right TPJ | 0.008 | 0.006 | -0.005 | 0.021 |  |  |  |
| R : right TPJ | 0.016 | 0.007 | 0.003 | 0.029 |  |  |  |

Table S3: Model 2 Contrasts for HbR values

|  | *Estimates* | *SE* | *CI Lower* | *CI Upper* | *t* | *p* |
| --- | --- | --- | --- | --- | --- | --- |
| Region = left dlPFC | | | | | | |
| C vs. FP | -0.025 | 0.009 | -0.051 | 0.000 | -2.68 | .057 |
| C vs. PD | -0.003 | 0.005 | -0.015 | 0.009 | -0.69 | .958 |
| C vs. PS | -0.007 | 0.004 | -0.020 | 0.006 | -1.46 | .582 |
| C vs. R | 0.043 | 0.012 | 0.011 | 0.075 | 3.62 | 0.003 |
| FP vs. PD | 0.022 | 0.010 | -0.006 | 0.050 | 2.12 | 0.209 |
| FP vs. PS | 0.018 | 0.009 | -0.006 | 0.042 | 2.06 | .235 |
| FP vs. R | 0.068 | 0.017 | 0.020 | 0.116 | 3.88 | .001 |
| PD vs. PS | -0.004 | 0.005 | -0.017 | 0.010 | -0.75 | .942 |
| PD vs. R | 0.046 | 0.010 | 0.017 | 0.073 | 4.33 | <.001 |
| PS vs. R | 0.050 | 0.013 | 0.014 | 0.085 | 3.84 | .001 |
| Region = right dlPFC | | | | | | |
| C vs. FP | -0.005 | 0.007 | -0.025 | 0.014 | -0.73 | .948 |
| C vs. PD | 0.001 | 0.005 | -0.013 | 0.015 | 0.25 | .999 |
| C vs. PS | -0.003 | 0.006 | -0.019 | 0.012 | -0.59 | .975 |
| C vs. R | 0.039 | 0.012 | 0.006 | 0.072 | 3.22 | .011 |
| FP vs. PD | 0.006 | 0.006 | -0.009 | 0.023 | 1.11 | .803 |
| FP vs. PS | 0.002 | 0.006 | -0.014 | 0.018 | 0.31 | .998 |
| FP vs. R | 0.044 | 0.012 | 0.012 | 0.077 | 3.73 | .002 |
| PD vs. PS | -0.004 | 0.005 | -0.017 | 0.008 | -1.02 | .843 |
| PD vs. R | 0.038 | 0.012 | 0.004 | 0.072 | 3.01 | .022 |
| PS vs. R | 0.042 | 0.013 | 0.006 | 0.078 | 3.25 | .010 |
| Region = left TPJ | | | | | | |
| C vs. FP | -0.008 | 0.008 | -0.032 | 0.015 | -0.95 | .873 |
| C vs. PD | -0.008 | 0.006 | -0.025 | 0.008 | -1.38 | .635 |
| C vs. PS | -0.006 | 0.006 | -0.023 | 0.012 | -0.88 | .904 |
| C vs. R | 0.038 | 0.012 | 0.004 | 0.072 | 3.07 | .018 |
| FP vs. PD | 0.000 | 0.007 | -0.021 | 0.020 | -0.05 | 1.00 |
| FP vs. PS | 0.002 | 0.005 | -0.016 | 0.021 | 0.36 | .984 |
| FP vs. R | 0.046 | 0.011 | 0.013 | 0.079 | 3.87 | .001 |
| PD vs. PS | 0.003 | 0.005 | -0.012 | 0.018 | 0.52 | .984 |
| PD vs. R | 0.046 | 0.013 | 0.009 | 0.084 | 3.41 | .006 |
| PS vs. R | 0.043 | 0.014 | 0.005 | 0.082 | 3.10 | .016 |
| Region = right TPJ | | | | | | |
| C vs. FP | -0.036 | 0.006 | -0.053 | -0.018 | -5.50 | <.001 |
| C vs. PD | -0.019 | 0.005 | -0.033 | -0.004 | -3.60 | .003 |
| C vs. PS | -0.015 | 0.005 | -0.028 | -0.001 | -2.98 | .024 |
| C vs. R | 0.026 | 0.012 | -0.007 | 0.061 | 2.11 | .213 |
| FP vs. PD | 0.016 | 0.007 | -0.003 | 0.036 | 2.36 | .126 |
| FP vs. PS | 0.020 | 0.006 | 0.003 | 0.038 | 3.25 | .010 |
| FP vs. R | 0.062 | 0.015 | 0.020 | 0.105 | 4.01 | .001 |
| PD vs. PS | 0.004 | 0.005 | -0.009 | 0.017 | 0.83 | .920 |
| PD vs. R | 0.045 | 0.014 | 0.007 | 0.084 | 3.62 | .010 |
| PS vs. R | 0.041 | 0.013 | 0.005 | 0.079 | 3.07 | .018 |

**Behavioral analyses**

We further analyzed the potential effect of concurrent movement, assessed by reaction time differences to the button press in the control and both explicit prediction conditions, mutual correct prediction of the same action (i.e., prediction accuracy), were related to neural synchrony. We, therefore, added the fixed effects of differences in reaction time to Model 2 and tested for the fixed effect and interaction effects with condition and region of interest.

wtc ~ condition * region * RT difference

However, the models displayed no significant relations of the aforementioned variables to neural synchrony, *p*>.201.

In a further analysis, we investigated the role of prediction accuracy on HbR synchrony in the Prediction-Same Action condition. We chose the amount of the same actions to mark prediction accuracy. We have used the following model for statistical tests (* indicates the inclusion of both fixed and interaction effects):

wtc ~ amount of same actions * roi + (1+amount of same actions*roi | dyad)

The results of the model showed no significant fixed effect of the same actions, p=.538 nor in interaction with region of interest, *p*=.558.


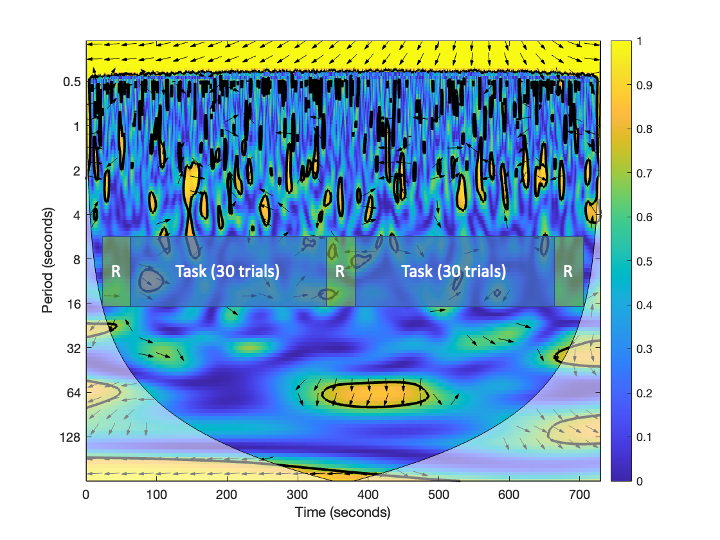


Figure S1. The graph depicts an exemplary WTC plot. Cycle period (in seconds) on the y axis and time (in seconds) on the x axis are used to extract coherence values for the three resting phases (R; green boxes) and the task phase (Task; blue boxes). The coherence value is averaged over the frequency/period band and the duration of the resting or task phase, respectively, thus resulting in a coherence value per box. For the statistical analyses, the coherence values were further averaged over the repetitions. All three green boxes, thus, resulted in one coherence value and both blue boxes were averaged, resulting in one coherence value.
